# Microbiota profile of new-onset celiac disease in children in Saudi Arabia

**DOI:** 10.1186/s13099-022-00493-1

**Published:** 2022-09-08

**Authors:** Mohammad El Mouzan, Abdulrahman Al-Hussaini, Gloria Serena, Asaad Assiri, Ahmed Al Sarkhy, Mohammad Al Mofarreh, Mona Alasmi, Alessio Fasano

**Affiliations:** 1 Department of Pediatrics (Gastroenterology), King Saud University, Riyadh, Kingdom of Saudi Arabia; 2 Department of Pediatrics, Children’s Specialist Hospital, King Fahad Medical City, Riyadh, Kingdom of Saudi Arabia; 3 Faculty of Medicine, AlFaisal University, Riyadh, Kingdom of Saudi Arabia; 4 Massachusetts General Hospital and Division of Pediatric Gastroenterology and Nutrition, Center for Celiac Research, Mucosal Immunology and Biology Research Center, Boston, MA USA; 5 Harvard Medical School, Boston, MA USA; 6 Al Mofarreh Polyclinic, King Fahad Medical City, Pediatric Intestinal Failure and Parenteral Nutrition Program, Riyadh, Kingdom of Saudi Arabia; 7 European Biomedical Research Institute of Salerno, Salerno, Italy

**Keywords:** Bacteriome, Celiac disease, Children

## Abstract

**Background:**

Intestinal dysbiosis has been reported to be associated with celiac disease (CeD) in Western populations but little is known in other populations who have different dietary lifestyle and genetic background. The purpose of this study was to determine whether a different microbiota profile is associated with CeD in children in Saudi Arabia.

**Results:**

Forty children with CeD, 20 healthy controls, and 19 non-CeD controls were enrolled. The median age at diagnosis was 10.3, 11.3 and 10.6 years in children with CeD, fecal, and mucosal control groups, respectively. Significant differences in microbial composition between children with CeD and controls both at fecal and mucosal level were identified. Fecal samples were more diverse and richer in bacteria as compared with mucosal samples. Proteobacteria were more abundant in duodenal mucosal samples and Firmicutes and Bacteroides were more abundant in stools. The abundance of many taxa was significantly different between children with CeD and non-CeD controls. In mucosal samples, *Bifidobacterium angulatum* (unadjusted p = 0.006) and *Roseburia intestinalis* (unadjusted p = 0.031) were examples of most significantly increased species in children with CeD and non-CeD controls, respectively. In fecal samples, there were 169 bacterial species with significantly different abundance between children with CeD and non- CeD controls.

**Conclusions:**

To our knowledge, this is the first report on the microbial profile in a non-Western population of children with new onset CeD. The fact that mucosal and fecal samples were collected from newly diagnosed children with CeD on normal gluten-containing diet suggests strong association between the identified bacteria and CeD. The identification of many unreported bacterial species significantly associated with CeD, indicates the need for further studies from different populations to expand our understanding of the role of bacteria in the pathogenesis of CeD, hopefully leading to the discovery of new adjuvant treatment options.

## Background

Celiac disease (CeD) is defined as an autoimmune enteropathy triggered by gluten, affecting genetically predisposed individuals (HLA DQ2 and/or DQ8) [1, 2]. Recent data show that tolerance to gluten can be lost at any time in life [3]. These findings, together with the lack of complete CeD concordance among monozygotic twins, suggest that, while genetic predisposition and gluten intake are necessary for CeD development, they are insufficient to trigger the onset of the disease [4]. Thus, other contributing factors such as changes in microbiome composition and function have been suggested to be associated with CeD.

The microbiome of a healthy individual is relatively stable by 3 years of age; however, this composition can be modulated throughout the entire lifespan by different factors, such as lifestyle, dietary choices, antibiotic treatment, stress, and other environmental components. Intestinal dysbiosis via such factors has been reported to be associated with development of CeD [5].

The Saudi population has been reported to have a high prevalence of CeD (1.5%). The high rate of CeD-predisposing HLA-DQ genotypes in the general population (52.7%) may partially account for this high prevalence, although additional external factors should also be taken into consideration [6]. The consumption of gluten-containing cereals in the diet of the Saudi population is reported to be very high as recorded by the Food and Drug Organization [7]. This high intake of cereals may directly increase the prevalence of CeD, or indirectly by altering other factors such as the microbiome composition.

Most of the literature on the microbiome in CeD were from Western populations. Cultural and dietary lifestyle in non-Western populations, mostly developing countries could affect microbiota profile and studies on microbiome in CeD from these populations may increase our understanding of the pathogenesis of CeD. Therefore, our objective was to determine whether a different microbiota profile is associated with CeD in children in Saudi Arabia.

## Results

### Characteristics of the study population

A total of 40 children with CeD (provided 20 tissue and 20 stool samples) and 39 controls were enrolled in this study. There were two types of controls. Twenty healthy children who provided stool samples only (fecal controls), and 19 non-CeD children who provided mucosal samples only (mucosal controls). The latter had normal endoscopy and normal duodenal mucosal histopathology. In addition, all controls had normal anti-tissue transglutaminase A values. The demographic and clinical characteristics are presented in Table 1. Briefly, males accounted for 28%, 35%, and 42% of the children with CeD, fecal, and mucosal control groups respectively. The median age at diagnosis was 10.3, 11.3 and 10.6 years in children with CeD, fecal, and mucosal control groups respectively. The number of asymptomatic children with CeD was 15/40 (38%), whereas the remainder had various combination of symptoms including anemia, growth impairment, and abdominal pain.

**Table 1:** Demographic and clinical characteristics

Variables	Celiac disease	Fecal controls	Mucosal controls
Number of children	40	20	19
Sex (% male sex)	28%	35%	42%
Age at presentation in years: median (range)	10.3 (7.5–15.7)	11.3 (6.8 -15.4)	10.6 (2–17.2)
Breastfeeding (%)		85%	68%
*Clinical presentation*			
Asymptomatic	15 (38%)	20 (100%)	0 (0%)
Anemia	11 (28%)	0 (0%)	1 (5%)
Diarrhea/A. distention	7 (18%)	0 (0%)	0 (0%)
Growth impairment	10 (25%)	0 (0%)	1 (5%)
Abdominal pain	10 (25%)	0 (0%)	10 (53%)
Constipation	8 (20%)	0 (0%)	0 (0%)
Vomiting	0 (0%)	0 (0%)	3 (16%)
Dysphagia	0 (0%)	0 (0%)	4 (21%)

### Alpha- and beta-diversities

**Fig. 1:**
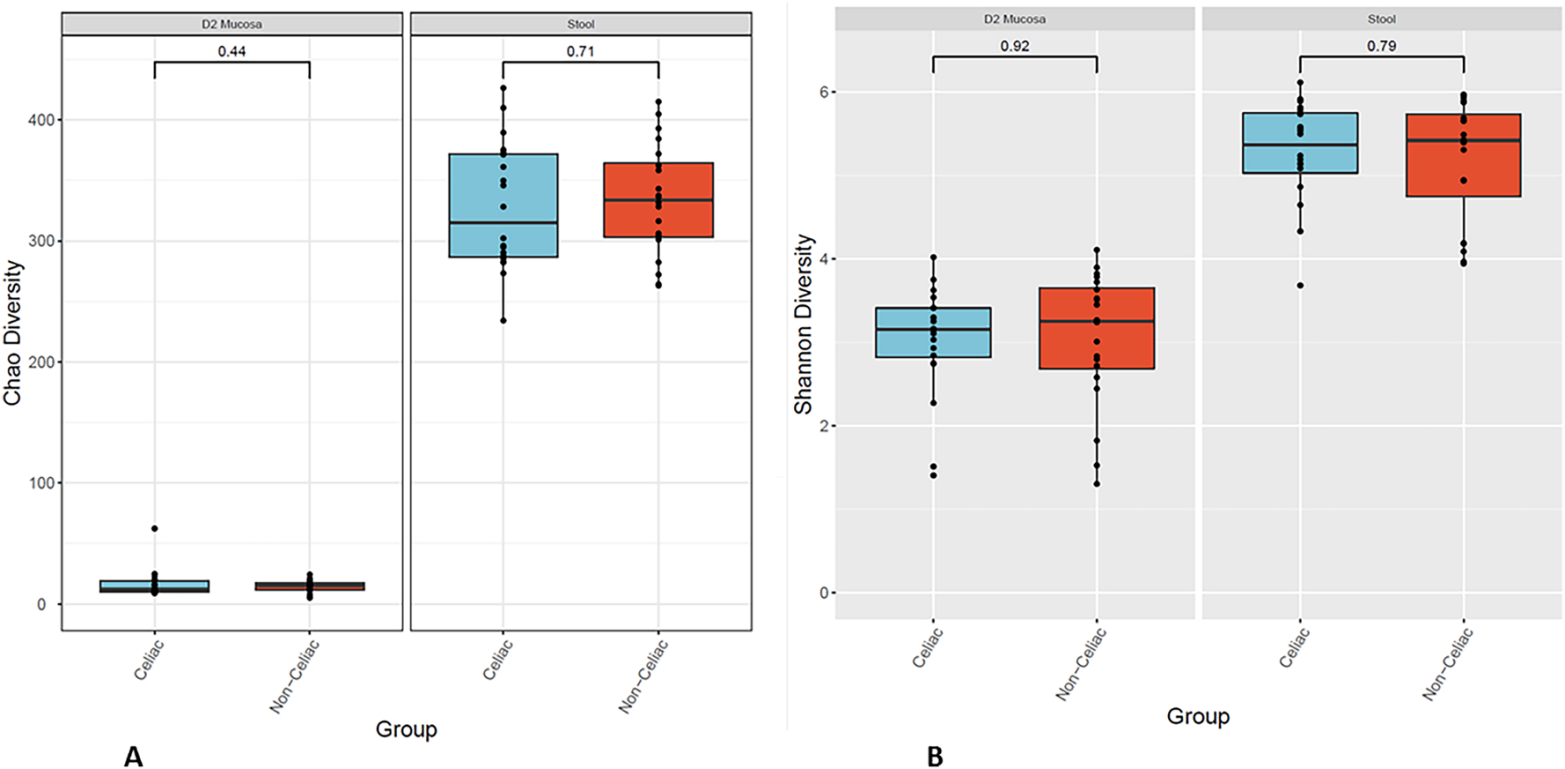
Alpha diversity. Illustration of alpha diversity measured by Chao index (**A**) and by Shannon index (**B**) for bacterial communities in duodenal and fecal samples of CeD patients and non-CeD controls

Differences in alpha diversity between the CeD and non-CeD groups were measured in both fecal and duodenal samples using the Chao and Shannon indices, an abundance-based estimators of species richness. Although not statistically significant, our analysis showed a clear difference in bacterial diversity between the mucosal and fecal samples, indicating an increased richness and variability in stools (Fig. 1A, B). Interestingly, alpha diversity did not differ between CeD and non-CeD groups, despite there being a trend toward smaller diversity in CeD stools compared with that in non-CeD stools.

**Fig. 2:**
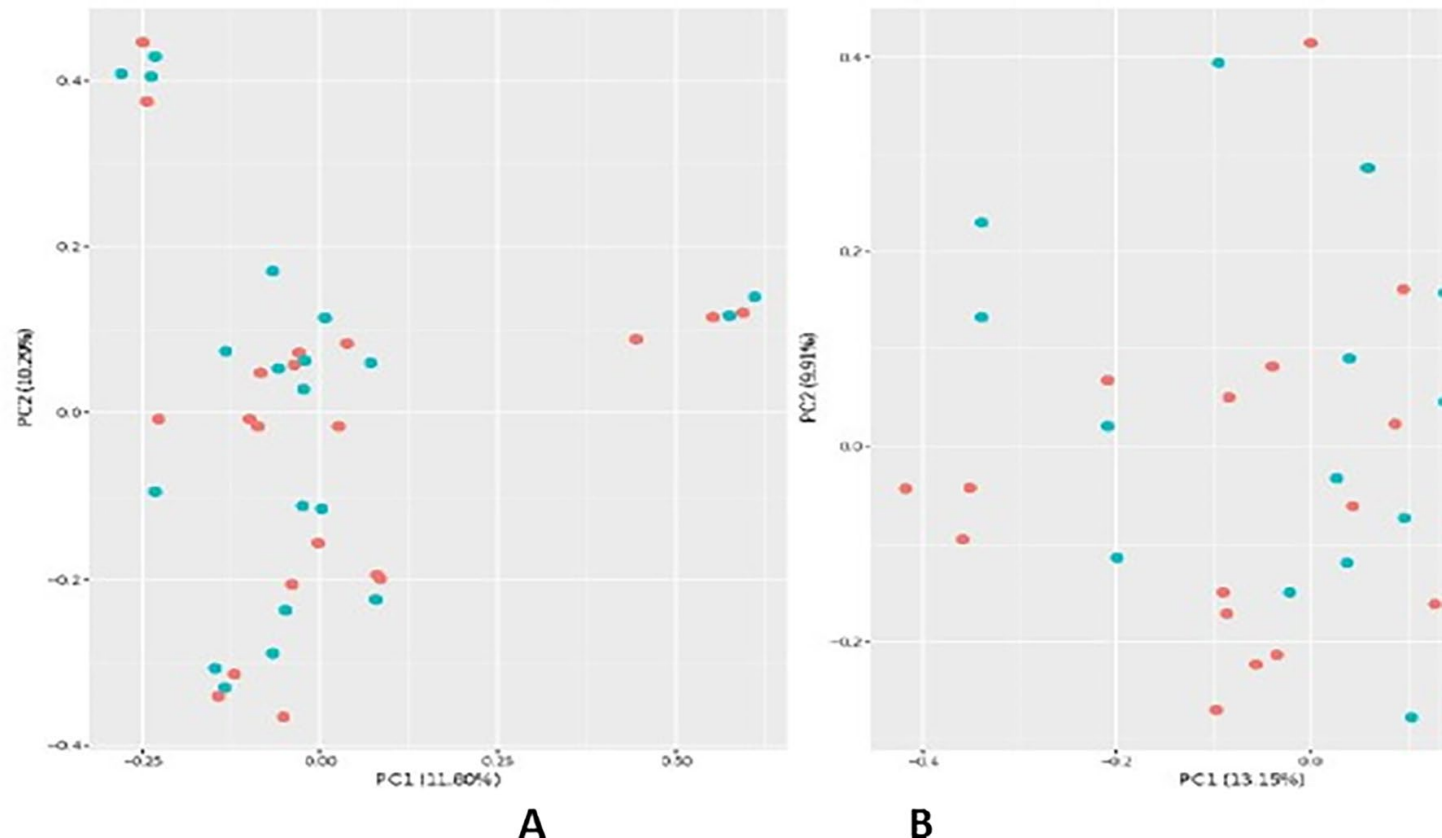
Beta diversity. Bray–Curtis-based bacterial beta-diversity analysis of mucosal (**A**) and fecal samples (**B**) from patients with CeD (pink dots) or non-CeD controls (blue dots)

For bacterial beta diversity, Bray–Curtis PCoA analysis did not show any significant clustering patterns in samples from the duodenal mucosa or stools of the CeD and non-CeD groups (Fig. 2A, B). However, in the analysis of bacterial fecal samples, there were small clusters characteristic of either CeD or non-CeD groups.

### Overall bacterial composition

**Fig. 3:**
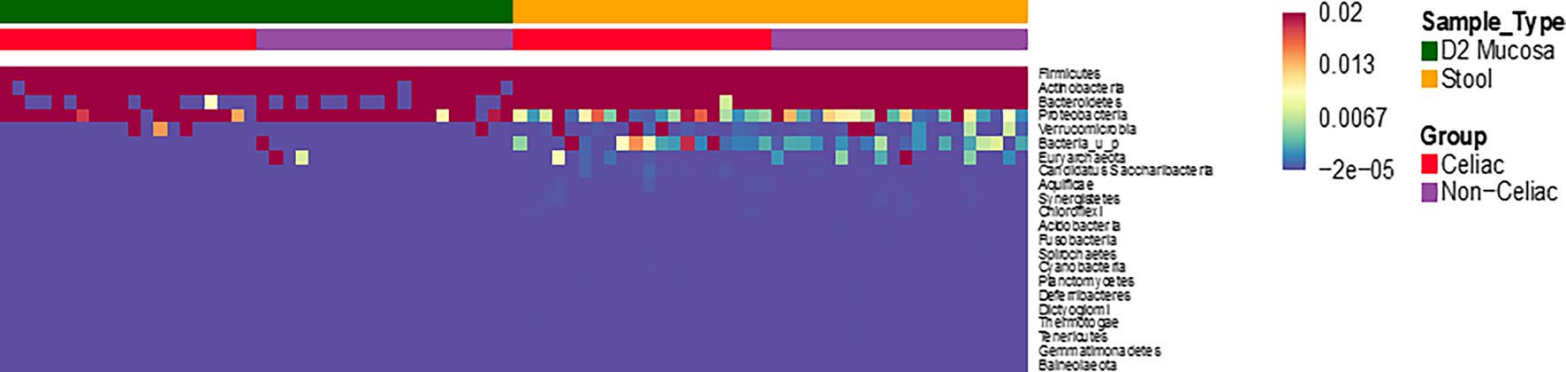
Heatmap: Representing bacterial microbiome composition in duodenal and fecal samples of patients with the CeD and non-CeD. The bacterial richness in stools was higher than that in duodenal samples, Firmicutes and Bacteroidetes were the most abundant phyla. Actinobacteria abundance was reduced

The overall bacterial composition of fecal and mucosal samples was analyzed in both CeD and non-CeD groups; this was represented through heatmap (Fig. 3). As expected, the bacterial richness in stools was higher than that in duodenal samples, and in both sets of samples, Firmicutes and Bacteroidetes were the most abundant phyla. In duodenal samples, an increased percentage of Proteobacteria species was detected, whereas overall, the stools were characterized by increased abundance of *Verrucomicrobia* species.

### LDA effect size

**Fig. 4:**
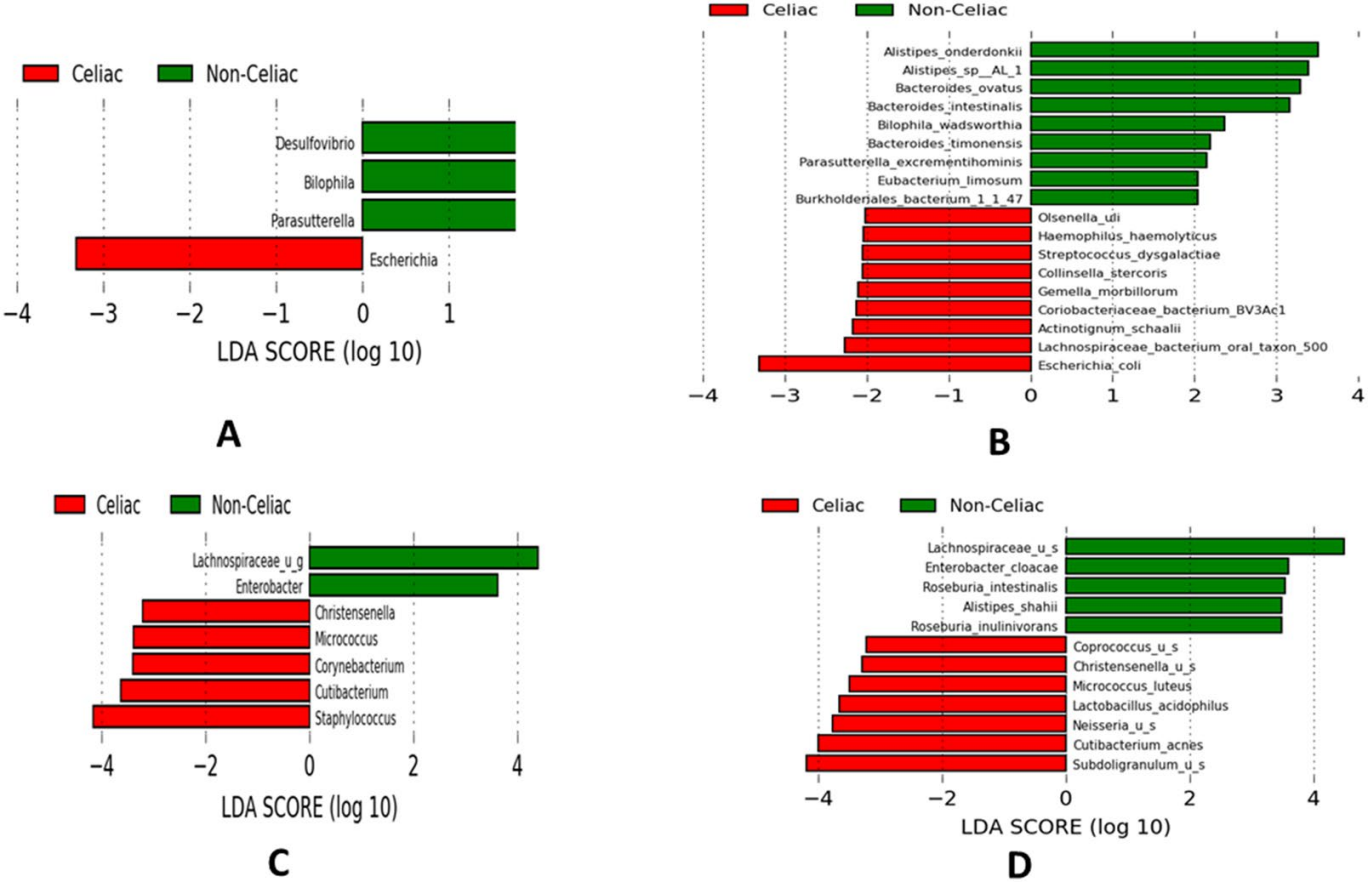
LEfSE LDA scores: **A** and **B** show statistically significant abundance difference in stool samples from patients with CeD with those of non-CeD controls at the genus (**A**) and species (**B**) level. **C** and **D** Illustrate the abundance difference, although not statistically different, of LDA scores in mucosal samples from patients with CeD with those of non-CeD controls at the genus (**C**) and species (**D**) level. Bars with a positive LDA score (green) are higher in non-celiac samples, and bars with a negative LDA score (red) are higher in celiac samples. The extensions u_g and _u_s mean unclassified genera and species respectively

The LDA effect size (LEfSe) plot revealed statistically significant different bacterial composition in fecal samples between children with CeD and non-CeD controls. For example, there was an increase of *Escherichia* in the CeD group and an increase of *Desulfovibrio* in the non-CeD group at the genus level (decreased in the CeD group) (Fig. 4A). Similarly, at the species level, there was a statistically-significant difference between the CeD and non-CeD group. For example, in the CeD group there was an increase of *E. coli* and *Lachnospiraceae_bacterium_oral*; whereas several species of *Bacteroides* were significantly increased in fecal samples of non-CeD controls (decreased in CeD) (Fig. 4B). In mucosal samples, although not statistically different by standard criteria, there were important differences in abundance of several taxa between CeD and non- CeD mucosal samples. For example, *Lactobacillus acidophilus, Neisseria* and *Coprococcus* species were increased in the CeD group; whereas *Roseburia* and *Lachnospiraceae* species were increased in non-CeD group (decreased in the CeD group) (Fig. 4C, D).

### DeSeq2 differential abundance analysis

DeSeq2 differential abundance analysis revealed statistically significant differences in log2 fold change abundance between CeD and non-CeD samples. Log2 fold change > 0 and < 0 indicate increased abundance in children with CeD and non-CeD respectively. Increased abundance in children with non-CeD suggests decreased abundance in children with CeD. Table 2 shows the log2 abundance change for the top 10 taxa (order, family, and genera) in mucosal and fecal samples, illustrating the different microbiota profile between mucosa and stool. For example, in mucosal samples, Flavobacteriales (p = 0.0005), Flavobacteriaceae (unadjusted p = 8.11^–08^), and *Clostridium* (unadjusted p = 0.011), were the most significantly decreased bacterial order, family, and genus levels respectively, whereas Micrococcales (unadjusted p = 0.018), Micrococcaceae (unadjusted p = 0.022), and *Subdoligranulum* (unadjusted p = 0.021) were the most significantly abundant bacterial order, family and genus levels respectively. In fecal samples, however, Cardiobacteriales (p = 0.01), Leuconostocaceae (p = 0.003), and *Tannerella* (p = 1.17^–05^) were the most significantly decreased bacterial order, family and genus levels respectively, whereas Planctomycetaceae (p = 0.013) and *Kocuria* (p = 0.003) were the most abundant family and genera levels. The top 10 most significant species abundance in mucosal samples is presented in Table 3. In these samples, *Bifidobacterium angulatum* (unadjusted p = 0.006) and *Roseburia intestinalis* (unadjusted p = 0.031), were examples of increased species in mucosal samples of children with CeD and non-CeD (decreased in CeD) respectively.

**Table 2:** Log2 abundance change for bacterial order, family, and genera in children with CeD

Level	Organism	Log2 abundance	p value	*p value	Organism	Log2 abundance	p value	*p value
*Mucosal samples*					*Fecal samples*			
Order	Flavobacteriales	– 0.4366	1.93^–05^	0.0005	Cardiobacteriales	– 0.4882	0.0001	0.01
Order	Micrococcales	1.0333	0.018	0.253	Methanobacteriales	– 1.3944	0.0004	0.02
Family	Flavobacteriaceae	– 0.3014	8.11^–08^	0.219	Leuconostocaceae	– 2.9810	4.20^–05^	0.003
Family	Clostridiaceae	– 1.0072	0.008	0.274	Cardiobacteriaceae	– 0.4851	0.0002	0.010
Family	Lactobacillaceae	– 0.0115	0.017	0.274	Planctomycetaceae	1.5023	0.0003	0.013
Family	Micrococcaceae	0.9708	0.022	0.219	Methanobacteriaceae	– 1.2405	0.0004	0.014
Genus	*Clostridium*	– 0.9103	0.011	0.486	*Tannerella*	– 2.6849	4.81^–08^	1.17^–05^
Genus	*Lactobacillus*	0.0586	0.015	0.486	*Citrobacter*	– 2.4701	7.65^–05^	0.003
Genus	*Subdoligranulum*	0.8853	0.021	0.486	*Methanobrevibacter*	– 1.4565	0.0007	0.017
Genus	*Kocuria*	0.7998	0.036	0.634	*Kocuria*	1.7515	9.25^–05^	0.003

*p-value adjusted for false discovery rate

**Table 3:** Log2 fold abundance change of the top 10 bacterial species in mucosal samples of children with CeD

Organism	Log 2-fold change	p value	*p value
*Bifidobacterium angulatum*	0.4177	0.006	0.460
*Lactobacillus acidophilus*	0.8935	0.011	0.460
*Kocuria rhizophila*	0.8462	0.021	0.460
*Roseburia intestinalis*	– 0.7395	0.031	0.460
*Ralstonia pickettii*	0.7255	0.037	0.460
*Acinetobacter lwoffii*	0.7059	0.042	0.460
*Corynebacterium ihumii*	0.6856	0.047	0.460
*Corynebacterium tuberculostearicum*	0.6856	0.047	0.460
*Bradyrhizobium sp. DFCI-1*	1.5177	0.075	0.601
*Staphylococcus aureus*	0.5959	0.0801	0.601

*p value adjusted for false discovery rate

The log2 fold change abundance of 169 significantly different bacterial species in fecal samples of children with CeD and non-CeD controls is depicted in Table 4. There were several species significantly decreased in children with CeD belonging to the *Bifidobacterium* genus, such as *B. breve* (p = 0.0028), *B. angolatum* (p = 2.24^−07^), *B. merycicum* (p = 0.012), and *B. thermophilum* (p = 0.027). Among *Lactobacilli* species, *L*. *plantarum* (p = 0.0043), was significantly less abundant in CeD samples, whereas the abundance of other lactobacilli such as *L. gasseri* (p = 0.033) was significantly- increased in children with CeD. *Prevotella* species (*P. timonensis (*p = 0.018); *P. bergensis* (p = 0.022) were significantly more abundant in stool samples of children with CeD, whereas *Prevotella sp. P5-119* was significantly less abundant (p = 1.69^–06^). Finally, several *Bacteroides* species were less abundant in fecal samples from children with CeD. In contrast, different *Clostridium* species were increased in abundance among children with CeD.

**Table 4:** Log2 fold change abundance of bacterial species in fecal samples of children with CeD

S. no.	Organism	Log2 fold change	P value	*p value
1	*Clostridium sp. L2-50*	– 2.0120	8.85^–20^	3.43^–17^
2	*Ruminococcus sp. SR1/5*	– 0.2670	9.89^–20^	3.43^–17^
3	*Streptococcus sp. SR4*	0.0787	2.91^–13^	6.75^–11^
4	*Actinomyces sp. oral taxon 175*	2.0207	1.80^–12^	3.12^–10^
5	*Clostridiales bacterium VE202-07*	0.7391	3.68^–11^	5.11^–09^
6	*Actinomyces sp. ICM39*	– 1.6899	1.01^–10^	1.16^–08^
7	*Bacteroides sp. 1_1_30*	– 0.9884	1.39^–10^	1.37^–08^
8	*Alistipes inops*	– 5.4881	4.50^–10^	3.90^–08^
9	*Desulfovibrio piger*	– 0.7593	7.71^–10^	5.94^–08^
10	*Actinomyces sp. ICM58*	– 5.2606	3.41^–09^	2.24^–07^
11	*Bifidobacterium angulatum*	– 1.3309	3.55^–09^	2.24^–07^
12	*Actinobaculum massiliense*	4.2776	6.03^–09^	3.49^–07^
13	*Bacteroides clarus*	– 3.5549	6.67^–09^	3.56^–07^
14	*Oscillibacter sp. KLE 1745*	1.8464	3.35^–08^	1.66^–06^
15	*Lactobacillus acidophilus*	0.8589	3.69^–08^	1.69^–06^
16	*Prevotella sp. P5-119*	– 3.6227	4.01^–08^	1.69^–06^
17	*Burkholderiales bacterium 1_1_47*	– 3.2591	4.14^–08^	1.69^–06^
18	*Coprobacter fastidiosus*	– 4.7198	6.62^–08^	2.55^–06^
19	*Tannerella sp. 6_1_58FAA_CT1*	– 2.9135	7.82^–08^	2.85^–06^
20	*Blautia hydrogenotrophica*	4.3470	1.11^–07^	3.88^–06^
21	*Coriobacteriaceae bacterium BV3Ac1*	2.8402	1.51^–07^	4.99^–06^
22	*Phascolarctobacterium succinatutens*	0.1041	2.10^–07^	6.63^–06^
23	*Citrobacter freundii*	– 3.9256	3.56^–07^	1.07^–05^
24	*Leuconostoc pseudomesenteroides*	– 4.7313	5.15^–07^	1.49^–05^
25	*Parasutterella excrementihominis*	– 2.8930	1.38^–06^	3.84^–05^
26	*Coprobacillus sp. D6*	0.7717	2.13^–06^	5.69^–05^
27	*[Ruminococcus] gnavus*	1.4824	2.73^–06^	7.03^–05^
28	*Klebsiella variicola*	– 3.4290	3.35^–06^	8.32^–05^
29	*Actinomyces sp. HPA0247*	0.3485	4.56^–06^	0.0001
30	*[Clostridium] spiroforme*	– 0.9840	8.71^–06^	0.0002
31	*Bacteroides eggerthii*	– 3.4963	1.24^–05^	0.0002
32	*Megasphaera massiliensis*	2.4114	2.15^–05^	0.0004
33	*Clostridiales bacterium VE202-26*	2.0418	2.29^–05^	0.0004
34	*Megasphaera sp. BL7*	– 3.4443	2.52^–05^	0.0005
35	*Actinotignum schaalii*	2.0700	3.82^–05^	0.0007
36	*Enterococcus avium*	0.8707	7.96^–05^	0.0015
37	*Eggerthella sp. 1_3_56FAA*	– 0.3357	8.57^–05^	0.0016
38	*Corynebacterium pyruviciproducens*	5.6575	8.87^–05^	0.0016
39	*Eubacterium limosum*	– 0.2074	0.0001	0.0018
40	*Bifidobacterium breve*	– 0.6821	0.0001	0.0028
41	*Mitsuokella jalaludinii*	0.7105	0.0001	0.0028
42	*Weissella confusa*	– 2.7298	0.0002	0.0037
43	*Streptococcus pneumoniae*	1.8511	0.0002	0.0042
44	*Lactobacillus plantarum*	– 1.0534	0.0002	0.0043
45	*Actinomyces cardiffensis*	– 0.1851	0.0004	0.0068
46	*Bacteroides faecichinchillae*	– 2.1328	0.0004	0.0068
47	*Lactobacillus mucosae*	0.6968	0.0005	0.0077
48	*Alistipes sp. HGB5*	0.9527	0.0005	0.0081
49	*Peptoniphilus harei*	2.6088	0.0006	0.0083
50	*Kocuria rhizophila*	1.5647	0.0006	0.0083
51	*Haemophilus haemolyticus*	1.8040	0.0007	0.0098
52	*Bacteroides gallinarum*	– 0.9100	0.0007	0.0098
53	*[Eubacterium] siraeum*	– 1.1823	0.0008	0.0103
54	*Scardovia inopinata*	2.6347	0.0008	0.0104
55	*Dialister succinatiphilus*	– 0.6384	0.001	0.011
56	*Eggerthella sp. YY7918*	– 2.9586	0.001	0.012
57	*Bifidobacterium merycicum*	– 1.2398	0.001	0.012
58	*Alistipes onderdonkii*	– 1.8638	0.001	0.012
59	*Lachnospiraceae bacterium oral taxon 500*	2.4268	0.001	0.013
60	*Alistipes sp. AL-1*	– 2.0624	0.001	0.013
61	*Kandleria vitulina*	– 1.1850	0.001	0.014
62	*Actinomyces turicensis*	1.7418	0.001	0.015
63	*Kallipyga massiliensis*	1.4601	0.001	0.015
64	*Prevotella timonensis*	2.3958	0.002	0.018
65	*Alistipes indistinctus*	– 0.9069	0.002	0.018
66	*Streptococcus parauberis*	– 1.4411	0.002	0.021
67	*Streptococcus dysgalactiae*	3.0429	0.002	0.021
68	*Acinetobacter junii*	1.3684	0.002	0.022
69	*Prevotella bergensis*	2.2901	0.002	0.022
70	*Anaerococcus prevotii*	1.3590	0.002	0.023
71	*Bifidobacterium biavatii*	0.1734	0.002	0.023
72	*Weissella cibaria*	– 3.1430	0.003	0.026
73	*Peptostreptococcus anaerobius*	1.7918	0.003	0.026
74	*Gardnerella vaginalis*	1.5445	0.003	0.026
75	*Eggerthia catenaformis*	– 1.3356	0.003	0.027
76	*Bifidobacterium thermophilum*	– 0.8901	0.003	0.027
77	*Bacteroides finegoldii*	– 2.9953	0.003	0.027
78	*Atopobium vaginae*	1.5527	0.003	0.028
79	*Mitsuokella multacida*	2.0038	0.003	0.028
80	*Anaerostipes sp. 3_2_56FAA*	– 4.0899	0.003	0.028
81	*Catonella morbi*	1.5910	0.003	0.028
82	*Ruminococcus gauvreauii*	1.5330	0.003	0.029
83	*Streptococcus anginosus*	1.5551	0.004	0.031
84	*Dialister invisus*	– 0.8902	0.004	0.031
85	*Dialister micraerophilus*	2.4775	0.004	0.032
86	*Sharpea azabuensis*	– 1.5192	0.004	0.033
87	*Lactobacillus gasseri*	2.1423	0.004	0.033
88	*Coprobacillus sp. D7*	0.5299	0.004	0.033
89	*Escherichia sp. 1_1_43*	0.9507	0.004	0.033
90	*Anaerococcus obesiensis*	1.2936	0.004	0.033
91	*Clostridium celatum*	1.5093	0.005	0.037
92	*Clostridium paraputrificum*	2.9282	0.005	0.037
93	*Actinomyces dentalis*	1.3394	0.005	0.038
94	*Coprococcus sp. HPP0074*	1.9454	0.005	0.038
95	*[Clostridium] saccharogumia*	1.2495	0.006	0.040
96	*Enterobacter cloacae complex 'Hoffmann cluster IV'*	1.3726	0.006	0.040
97	*Streptococcus sinensis*	1.2504	0.006	0.042
98	*Mageeibacillus indolicus*	– 1.2756	0.007	0.047
99	*Bacteroides stercorirosoris*	– 1.5318	0.007	0.048
100	*Bifidobacterium saguini*	2.2018	0.007	0.048
101	*Lactobacillus ultunensis*	2.0939	0.008	0.048
102	*Klebsiella sp. 10,982*	– 1.2026	0.008	0.048
103	*Klebsiella michiganensis*	5.7397	0.008	0.048
104	*Megasphaera micronuciformis*	– 0.3100	0.008	0.050
105	*Anaeroglobus geminatus*	2.7566	0.009	0.054
106	*Corynebacterium pseudodiphtheriticum*	1.1915	0.009	0.055
107	*Clostridiales bacterium VE202-09*	– 3.922	0.010	0.059
108	*Christensenella minuta*	– 0.3566	0.010	0.059
109	*Leuconostoc mesenteroides*	0.0528	0.010	0.061
110	*Bacteroides barnesiae*	– 2.0367	0.011	0.062
111	*Corynebacterium argentoratense*	– 0.7247	0.012	0.071
112	*Intestinimonas butyriciproducens*	0.3570	0.013	0.075
113	*Propionibacterium acidifaciens*	1.9602	0.014	0.079
114	*Klebsiella pneumoniae*	– 1.3439	0.016	0.084
115	*Streptococcus constellatus*	0.6456	0.017	0.095
116	*Anaerococcus vaginalis*	1.0155	0.018	0.097
117	*Enorma massiliensis*	1.2870	0.018	0.097
118	*Lachnospiraceae bacterium 2_1_58FAA*	1.4788	0.019	0.099
119	*Ruminococcaceae bacterium D16*	– 0.8005	0.020	0.105
120	*Bacteroides salanitronis*	0.0412	0.020	0.105
121	*Akkermansia muciniphila*	– 0.4313	0.021	0.111
122	*Bifidobacterium minimum*	– 2.4337	0.022	0.112
123	*Candidatus Saccharibacteria oral taxon TM7x*	1.1047	0.022	0.112
124	*Shigella sp. SF-2015*	1.7508	0.024	0.119
125	*Bifidobacterium callitrichos*	– 0.0551	0.024	0.119
126	*Lachnospiraceae bacterium 1_4_56FAA*	– 0.3516	0.025	0.124
127	*Bifidobacterium gallinarum*	0.2816	0.026	0.126
128	*Lachnospiraceae bacterium 9_1_43BFAA*	2.1702	0.027	0.128
129	*Turicibacter sp. HGF1*	1.9376	0.027	0.128
130	*Leucobacter chironomi*	– 0.9613	0.027	0.128
131	*Slackia exigua*	1.4279	0.027	0.128
132	*Streptococcus pyogenes*	1.9995	0.028	0.128
133	*Bulleidia extructa*	1.0910	0.028	0.129
134	*Streptococcus mutans*	1.2386	0.028	0.130
135	*bacterium LF-3*	1.1778	0.030	0.135
136	*Morococcus cerebrosus*	0.9367	0.030	0.136
137	*Klebsiella oxytoca*	1.8890	0.030	0.136
138	*Raoultella ornithinolytica*	– 4.1641	0.031	0.137
139	*Enterococcus saccharolyticus*	0.1371	0.031	0.137
140	*Mucispirillum schaedleri*	– 1.0032	0.032	0.138
141	*Butyricimonas synergistica*	0.1619	0.032	0.138
142	*Eubacterium ramulus*	– 0.9600	0.033	0.142
143	*Prevotella sp. BV3P1*	4.9067	0.034	0.144
144	*Parabacteroides goldsteinii*	1.2363	0.035	0.150
145	*Haemophilus pittmaniae*	0.2372	0.036	0.152
146	*Oribacterium sinus*	0.9714	0.036	0.152
147	*Pseudoramibacter alactolyticus*	0.1447	0.037	0.153
148	*Olsenella profusa*	2.2762	0.038	0.156
149	*Bacteroides pyogenes*	– 4.6019	0.038	0.158
150	*Streptococcus sp. SR1*	0.9154	0.039	0.160
151	*candidate division TM7 single-cell isolate TM7b*	1.1954	0.040	0.161
152	*Eikenella corrodens*	– 0.9100	0.041	0.165
153	*Enterobacter sp. MGH 38*	– 5.7753	0.042	0.165
154	*Streptococcus oralis*	1.0494	0.042	0.165
155	*Lactobacillus casei group*	– 0.8741	0.043	0.168
156	*Desulfovibrio desulfuricans*	– 1.1163	0.043	0.169
157	*Escherichia fergusonii*	– 1.4098	0.044	0.169
158	*Paraclostridium bifermentans*	0.9890	0.044	0.169
159	*Citrobacter koseri*	0.8884	0.045	0.169
160	*Granulicatella elegans*	– 0.3866	0.045	0.169
161	*Succinivibrio dextrinosolvens*	– 2.7856	0.046	0.171
162	*Streptococcus thermophilus*	– 0.8128	0.046	0.171
163	*Prevotella buccae*	0.8820	0.046	0.171
164	*Lactobacillus jensenii*	– 0.4273	0.046	0.171
165	*butyrate-producing bacterium SM4/1*	– 2.8000	0.047	0.171
166	*Atopobium sp. ICM42b*	– 0.9230	0.047	0.171
167	*Bifidobacterium longum*	0.9381	0.048	0.176
168	*Parabacteroides johnsonii*	1.6349	0.049	0.176
169	*Collinsella intestinalis*	1.0310	0.049	0.177

*p value adjusted for false discovery rate

## Discussion

The association between CeD and intestinal dysbiosis has already been described in several studies [8–11]. However, the exact role of the microbiome in CeD pathogenesis has not yet been fully elucidated, and, given the fundamental functions that the intestinal microbiota plays in regulating intestinal homeostasis, it has been suggested that specific changes in microbiome composition may contribute to CeD onset [12]. The intestinal microflora is very functionally diverse, and its composition can depend on the intestinal site considered [13, 14]. CeD is a duodenum-specific enteropathy, and changes in the small intestinal microbiome are therefore thought to be associated with its development [15]. However, several studies have also shown that patients with CeD present fecal microbiota dysbiosis [16]. These data suggest that, along with the small intestine, other parts of the gastrointestinal tract, such as the colon, may be a source of information for CeD pathogenesis.

This report, the first metagenomic analysis from a population of Saudi children, highlights several important differences between mucosal and fecal microbiome. Alpha-diversity analysis, for example, confirmed previously reported findings with fecal samples having increased bacterial richness and diversity as compared with those from mucosal samples [16]. Interestingly, we did not see any differences in alpha diversity between CeD and non-CeD groups. Microbial diversity in patients with CeD has been shown to be reduced compared with that in non-CeD controls [17], although another study found this was not the case [18]. Our analysis included a relatively small number of samples, which could account for the lack of significant differences in microbial diversity.

LDA LEfSe and DeSeq2 differential abundance analyses demonstrated significant differences between CeD and non-CeD groups at both mucosal and fecal levels. Overall, samples from patients with CeD appeared to have a decreased abundance of Actinobacteria phylum that is mainly represented by bacteria belonging to the *Bifidobacterium* genus. Many *Bifidobacteria* have positive immunomodulatory functions and are therefore used as probiotics. However, the increased abundance of *L. acidophilus* and *Coprococcus* species in in children with CeD contrasts with previous reports description as “good bacteria” [19, 20]. Samples from non-CeD controls appeared to have an increased abundance of “beneficial” bacteria (decreased in CeD) such as *Roseburia* and *Lachnospiraceae* species. *Roseburia* species are short-chain fatty acid-producing bacteria, which modulate intestinal motility and have anti-inflammatory properties. Changes in *Roseburia* species abundance have been correlated to several diseases such as irritable bowel syndrome, obesity, and type 2 diabetes [21, 22]. Similarly, *Lachnospiraceae* are often used as probiotics because of their “beneficial” impact on overall intestinal health [23]. Finally, increased levels of *Subdoligranulum* species have been found in CeD samples by several groups [18, 24]. Interestingly, a recent work by Leonard et al. demonstrated an increase in this specific genus in fecal samples from infants genetically predisposed to CeD even before the onset of the disease [24]. These findings are intriguing as they suggest a causative link between dysbiosis and CeD onset. Furthermore, they also raise the possibility that fecal microbiome markers could be representative of small intestinal dysbiosis. While our findings partially confirm previously reported differences between patients with CeD and those without, the use of metagenomic technology in this study revealed many unreported species, with significantly different abundance between children with CeD and non-CeD controls. Finally, it should be noted that bacterial associations with CeD reported in this study do not imply causality, a limitation that is common to most microbiota studies.

### Study limitations

The most important limitation of this study is the relatively small sample size. However, the use of shotgun metagenomic analysis and the finding of many unreported bacterial species in this population of Saudi Arab children with high prevalence CeD make the results unique. Other limitations included the unavailability of information on the diet, growth and results of laboratory investigations.

## Conclusions

Although preliminary, our data from Saudi Arabia, reports new bacterial species significantly associated with CeD. The fact that mucosal and fecal samples were collected from newly diagnosed children with CeD on normal gluten-containing diet suggests strong association between the identified bacteria and CeD. In addition, the identification of many unreported taxa associated with celiac disease, indicates the need for further studies from different populations to expand our understanding of the role of bacteria in the pathogenesis of celiac disease, hopefully leading to new treatment options.

## Methods

### Study population

The participants were enrolled from King Khalid University Hospital, King Saud University (KSU), Al Mofarreh PolyClinic, and King Fahad Medical City Children’s Hospital, Ministry of Health. All institutions are in Riyadh, Kingdom of Saudi Arabia (KSA). Main inclusion criteria included children below 18 years of age who were on normal gluten containing diet and had no history of antibiotic intake for at least 6 months before presentation to the clinic. In addition, confirmation of CeD for cases and exclusion of CeD for controls were according to the European Society of Pediatric Gastroenterology Hepatology and Nutrition guidelines [25].

### Samples collection, storage, and processing

Mucosal samples from 20 children with confirmed CeD and 19 non-CeD controls were collected from the second part of the duodenum (D2); these were then stored in cryovials without fixative or stabilizer and transported in ice to the Central Laboratory. Similarly, fecal samples were also collected in cryovials from 20 children with CeD and 20 non-CeD controls and transported in ice to the Central Laboratory at the College of Medicine, (KSU). All samples were stored at − 80 °C. At the time of analysis, all samples were retrieved and dispatched by express mail in dry ice containers with temperature control for metagenomic analysis at Cosmos ID (Rockville, MD, USA).

### DNA isolation and sequencing

DNA was isolated from mucosa samples using the ZymoBiomics miniprep kit and from stool samples using QIAGEN DNeasy PowerSoil DNA kit, both according to the manufacturer’s instructions. Isolated DNA was quantified via Qubit ds DNA HS assay kit (Thermo Fisher).

DNA libraries were prepared using the Illumina Nextera XT library preparation kit, according to the manufacturer’s protocol. Library quantity and quality were assessed using Qubit and TapeStation (Agilent Technologies, CA, USA). Libraries were then sequenced on Illumina HiSeq platform (2 × 150 bp reads). The samples were sequenced on the deeper end. They were sequenced at an average of about 20 million total reads per sample.

### Bioinformatic analysis

Unassembled sequencing reads were directly analyzed using CosmosID bioinformatics platform (CosmosID Inc., Rockville, MD) for multi-kingdom microbiome analysis and quantification of organism’s relative abundance [26–29]. Briefly, the system utilizes curated genome databases and a high-performance data mining algorithm that rapidly disambiguates hundreds of millions of metagenomic sequence reads into discrete microorganisms that engender the particular sequences.

### Custom analysis

#### Alpha-diversity boxplots

Alpha-diversity boxplots were calculated from the species-level abundance score matrices from CosmosID taxonomic analysis. Chao’s and Shannon’s alpha-diversity metrics were calculated in R using the R package Vegan. Further, t-tests were performed between each celiac and non-celiac group using the R package ggsignif. Boxplots with overlaid significance in p-value format were generated using the R package ggplot2 [30–32].

#### Beta-diversity principal coordinate analyses (PCoA)

Beta-diversity PCoA were calculated from the species-level relative abundance matrices from CosmosID taxonomic analysis. Bray–Curtis diversity was calculated in R using the R package Vegan with the functions vegdist; then, PCoA tables were generated using Vegan’s function PCoA. Plots were visualized using the R package ggpubr [30, 33].

#### Linear discriminant analysis effect size (LEfSe)

The LEfSe figures were generated using the Galaxy web application, based on relative abundance tables from CosmosID taxonomic analysis. Figures were calculated using a Kruskal–Wallis *P*-value of < 0.05, a Wilcoxon *P*-value of < 0.05, and a logarithmic linear discriminant analysis (LDA) score of ≥ 2.0 and therefore exhibited a statistically significant difference between groups. In addition, although not showing significant difference, some organisms may be functionally important. To explore this possibility, the P- values were set to < 0.2 for both Wilcoxon and Kruskal–Wallis tests, and the logarithmic LDA score of ≥ 0.05 and figures were calculated based on this threshold. In the LEfSe figures, the red bars (negative bars) indicate that the organism is more abundant in the CeD group; Whereas green bars ( positive bars) indicate greater organism abundance in the non-CeD group [34].

#### DeSeq2 differential abundance analysis

Differential abundance analysis used the abundance score matrices from the CosmosID taxonomic analysis. Differential abundance for organisms was calculated using DeSEQ2 from the R Phyloseq package (R Foundation for Statistical Computing, Vienna, Austria). For the mucosal and stool samples separately, the log2 fold change and associated P-values for celiac vs. non-celiac samples are displayed [35, 36]. A log2 > 0 indicates that the organism is more abundant in the CeD group; whereas a value < 0 indicates more abundance in the non-CeD group. P-values were calculated using the t-test function in R and adjusted for false discover rate. However, we also reported unadjusted p values to detect taxa not reaching the adjusted significance level but with possible biologic importance. The difference in abundance was considered significant when the adjusted P- value was < 0.05. In addition, unadjusted P- value was reported to reveal taxa that might have functional properties.

## Data Availability

The datasets generated and/or analyzed during the current study are available in the NCBI SRA. Access link: http://www.ncbi.nlm.nih.gov/bioproject/757365
